# Supplementary material for: ExTrack characterizes transition kinetics and diffusion in noisy single-particle tracks
Source: J Cell Biol. 2023 Mar 1;222(5):e202208059. doi: 10.1083/jcb.202208059 (PMC9997658; doi:10.1083/jcb.202208059)
Supplement: Table S1 — ExTrack fits of three-state models with different simulations of three-state data with qualitatively different types of transitions. [file JCB_202208059_TableS1.docx]

| 3-state models | | *σ* | *d*_0_ | *d*_1_ | *d*_2_ | *F*_0_ | *F*_1_ | *F*_2_ | *k*_01_ | *k*_02_ | *k*_10_ | *k*_12_ | *k*_20_ | *k*_21_ |  |
| --- | --- | --- | --- | --- | --- | --- | --- | --- | --- | --- | --- | --- | --- | --- | --- |
| 1 | True | 0.020 | 0.000 | 0.040 | 0.100 | 0.333 | 0.333 | 0.333 | 0.100 | 0.100 | 0.100 | 0.100 | 0.100 | 0.100 |  |
|  | Estimated | 0.020 | 0.001 | 0.040 | 0.101 | 0.342 | 0.330 | 0.328 | 0.097 | 0.093 | 0.098 | 0.093 | 0.098 | 0.098 |  |
| 2 | True | 0.020 | 0.000 | 0.000 | 0.140 | 0.069 | 0.517 | 0.414 | 0.300 | 0.000 | 0.000 | 0.040 | 0.050 | 0.000 |  |
|  | Estimated | 0.020 | 0.000 | 0.000 | 0.141 | 0.069 | 0.518 | 0.413 | 0.297 | 0.005 | 0.000 | 0.040 | 0.050 | 0.000 |  |
| 3 | True | 0.020 | 0.000 | 0.000 | 0.140 | 0.077 | 0.462 | 0.462 | 0.000 | 0.300 | 0.050 | 0.000 | 0.000 | 0.050 |  |
|  | Estimated | 0.020 | 0.000 | 0.000 | 0.141 | 0.089 | 0.494 | 0.417 | 0.075 | 0.190 | 0.047 | 0.009 | 0.000 | 0.051 |  |
| 4 | True | 0.020 | 0.000 | 0.000 | 0.140 | 0.060 | 0.489 | 0.451 | 0.250 | 0.050 | 0.020 | 0.040 | 0.040 | 0.010 |  |
|  | Estimated | 0.020 | 0.000 | 0.000 | 0.141 | 0.046 | 0.504 | 0.450 | 0.358 | 0.046 | 0.000 | 0.041 | 0.042 | 0.009 |  |

Supplementary Table 1. Here, we use ExTrack to ﬁt parameters of a three-state model to different simulations of three-state data with qualitatively different types of transitions. Model 1: immobile state, intermediate diffusion state and high diffusion state. 10,000 tracks of 10 positions. Models 2 to 4 represent harder models with two immobile states and one diffusive state (10,000 tracks of 50 positions each). They all have a transient immobile state (state 0), a stable immobile state (state 1), and a diffusive state (state 2). Model 2: transitions from state 0 to 1 to 2 to 0 in a circular fashion. Model 3: transitions from state 2 to 1 to 0 to 2 in a circular fashion. Model 4: more complex transitions between states.
