# Supplementary material for: ExTrack characterizes transition kinetics and diffusion in noisy single-particle tracks
Source: J Cell Biol. 2023 Mar 1;222(5):e202208059. doi: 10.1083/jcb.202208059 (PMC9997658; doi:10.1083/jcb.202208059)
Supplement: Table S2 — Two-state and three-state fits of tracks from simulated particles either in immobile state (state 0) or in one of five diffusive states (states 1–5). [file JCB_202208059_TableS2.docx]

| ExTrack Model | Set of *d* | *k_ij_* | Parameters | *σ* | *d_0_* | *d*_1_ | | *F*_0_ | *k*_u_^*^ | *k*_b_^*^ |
| --- | --- | --- | --- | --- | --- | --- | --- | --- | --- | --- |
| 2 states | (0.04, 0.06, 0.08, 0.10, 0.12) | 0.10 | Estimated | 0.021 | 0.001 | 0.090 | | 0.458 | 0.130 | 0.110 |
|  | |  | True | 0.020 | 0.000 | - | | 0.375 | 0.1 | 0.06 |
|  |  |  |  |  |  |  |  |  |  |  |
|  |  |  |  |  |  |  |  |  |  |  |
|  |  |  |  |  |  |  |  |  |  |  |
| ExTrack Model | Set of *d* | *k_ij_* | Parameters | *σ* | *d*_0_ | *d*_1_ | *d_2_* | *F*_0_ | *k*_u_^*^ | *k*_b_^*^ |
| 3 states | (0.04, 0.06, 0.08, 0.10, 0.12) | 0.00 | Estimated | 0.020 | 0.001 | 0.054 | 0.107 | 0.395 | 0.101 | 0.066 |
|  |  | 0.02 | Estimated | 0.020 | 0.001 | 0.053 | 0.107 | 0.387 | 0.101 | 0.064 |
|  |  | 0.10 | Estimated | 0.020 | 0.000 | 0.053 | 0.110 | 0.399 | 0.102 | 0.068 |
|  | (0.04, 0.08, 0.12, 0.16, 0.20) | 0.02 | Estimated | 0.020 | 0.000 | 0.064 | 0.174 | 0.405 | 0.105 | 0.072 |
|  |  |  | True | 0.020 | 0.000 | - | - | 0.375 | 0.100 | 0.060 |

Supplementary Table 2. 2-state and 3-state ﬁts of tracks from simulated particles either in immobile state (state 0) or in one of 5 diffusive states (states 1 to 5). Here, unbinding rates 𝑘0,𝑗 = 0.02 Δ𝑡^−1^, binding rates 𝑘0,𝑗 = 0.06 Δ𝑡^−1^ and other rates 𝑘𝑖,𝑗 = 0, 0.02 or 0.1 Δ𝑡^−1^ for 𝑖 and 𝑗 from 1 to 5. *, 𝑘u and 𝑘b are the global unbinding and binding rates, respectively, obtained as the sum of the unbinding rates, and the average of the binding rates weighted by the fractions in diffusive state. ExTrack settings: window length = 6. All distances in µm and rates in Δ𝑡^−1^
